# Mechanisms Underlying Biological Effects of Cruciferous Glucosinolate-Derived Isothiocyanates/Indoles: A Focus on Metabolic Syndrome

**DOI:** 10.3389/fnut.2020.00111

**Published:** 2020-09-02

**Authors:** Montserrat Esteve

**Affiliations:** ^1^Department of Biochemistry and Molecular Biomedicine, University of Barcelona, Barcelona, Spain; ^2^Biomedical Research Centre in Physiopathology of Obesity and Nutrition (CIBERobn), Institute of Health Carlos III, Madrid, Spain

**Keywords:** cruciferous, glucosinolates, isothiocyanates, indoles, metabolic syndrome, inflammation, Nuclear erythroid 2-related factor (Nrf2), Nuclear Factor-κB (NF-κB)

## Abstract

An inverse correlation between vegetable consumption and the incidence of cancer has long been described. This protective effect is stronger when cruciferous vegetables are specifically consumed. The beneficial properties of vegetables are attributed to their bioactive components like fiber, antioxidants vitamins, antioxidants, minerals, and phenolic compounds. Cruciferous vegetables contain all these molecules; however, what makes them different are their sulfurous components, called glucosinolates, responsible for their special smell and taste. Glucosinolates are inactive biologically in the organism but are hydrolyzed by the enzyme myrosinase released as a result of chewing, leading to the formation of active derivatives such as isothiocyanates and indoles. A considerable number of *in vitro* and *in vivo* studies have reported that isothiocyanates and indoles elicit chemopreventive potency through multiple mechanisms that include modulation of phases I and II detoxification pathway enzymes, regulation of cell cycle arrest, and control of cell growth, induction of apoptosis, antioxidant activity, anti-angiogenic effects, and epigenetic regulation. Nuclear erythroid 2-related factor 2 (Nrf2) and Nuclear factor-κB (NF-κB) are key and central regulators in all these processes with a main role in oxidative stress and inflammation control. It has been described that isothiocyanates and indoles regulate their activity directly and indirectly. Today, the metabolic syndrome (central obesity, insulin resistance, hyperlipidemia, and hypertension) is responsible for a majority of deaths worldwide. All components of metabolic syndrome are characterized by chronic inflammation with deregulation of the PI3K/AKT/mTOR, MAPK/EKR/JNK, Nrf2, and NF-κB signaling pathways. The effects of GLSs derivatives controlling these pathways have been widely described in relation to cancer. Changes in food consumption patterns observed in the last decades to higher consumption of ultra-processed foods, with elevation in simple sugar and saturated fat contents and lower consumption of vegetables and fruits have been directly correlated with metabolic syndrome prevalence. In this review, it is summarized the knowledge regarding the mechanisms by which cruciferous glucosinolate derivatives (isothiocyanates and indoles) directly and indirectly regulate these pathways. However, the review places a special focus on the knowledge of the effects of glucosinolates derivatives in metabolic syndrome, since this has not been reviewed before.

## Introduction

The aim of the present review was to assess the current knowledge about the mechanisms through which isothiocyanates (ITCs) and indoles derived from glucosinolates (GLSs) yield their biological effects. The effects to GLSs derivatives related to cancer have been extensively reviewed but have not been reviewed in relation to metabolic syndrome (MetS), which is what the review is focused on. Presented first are the factors that influence the amount of GLSs consumed, such as plant concentrations conditioned by growing, storage, and cooking conditions. Further is explained the process by which they are absorbed and metabolized. Then, the known biological effects and mechanisms through which ITCs and indoles act are assessed. It should be noted that the effects of ITCs and indoles have been studied mainly in relation to cancer, both in cancer cell lines and in cancer studies of animal models. However, it is necessary to point out that not only the anticancer effects have been analyzed; their effects in other situations are also mentioned because the purpose was to highlight the biological mechanisms they regulate, which can also be altered in other pathologies. Finally, it is focus on the metabolic MetS (which is greatly influenced by diet and is a major cause of death today) and analyzed the protective effects of ITCs and indoles brought about by the regulation of pathways previously described in relation to cancer prevention.

High consumption of vegetables and fruit has been recommended widely for the primary prevention of major chronic diseases such as type 2 diabetes (1, 2), coronary heart disease (3, 4), and some cancers such as those of the esophagus, larynx, stomach, colon and rectum, breast, lung, and bladder (5, 6). However, not all studies have yielded consistent results (7, 8). Some data show that not all vegetables have equal protective efficiency. Schulz et al. (9) found no evidence of an inverse linear association between total fruit and vegetable intake and the risk of ovarian cancer, but found that the consumption of garlic/onion might exert a protective effect on the risk of ovarian cancer. In a large study of men and women in the United States, Bhupathiraju et al. (4) suggested that the absolute quantity of fruits and vegetable consumed (rather than the variety) is associated with a significantly lower risk of coronary heart disease. However, the analysis of the correlation between the variety and quantity showed that higher intakes of specific fruits (such as citrus fruits), vegetables rich in β-carotene or vitamin C, and green leafy vegetables were associated with the lowest risk of coronary heart disease. In another study, Bazzano et al. (1) found that the consumption of green leafy vegetables and whole fruit was associated with a lower hazard of diabetes. Thus, the diverse protective effects of vegetables in relation to chronic diseases could be attributed to the differences in the composition of micronutrients and phytochemical profiles, cooking methods, and individual genetic variability.

**Figure 1:**
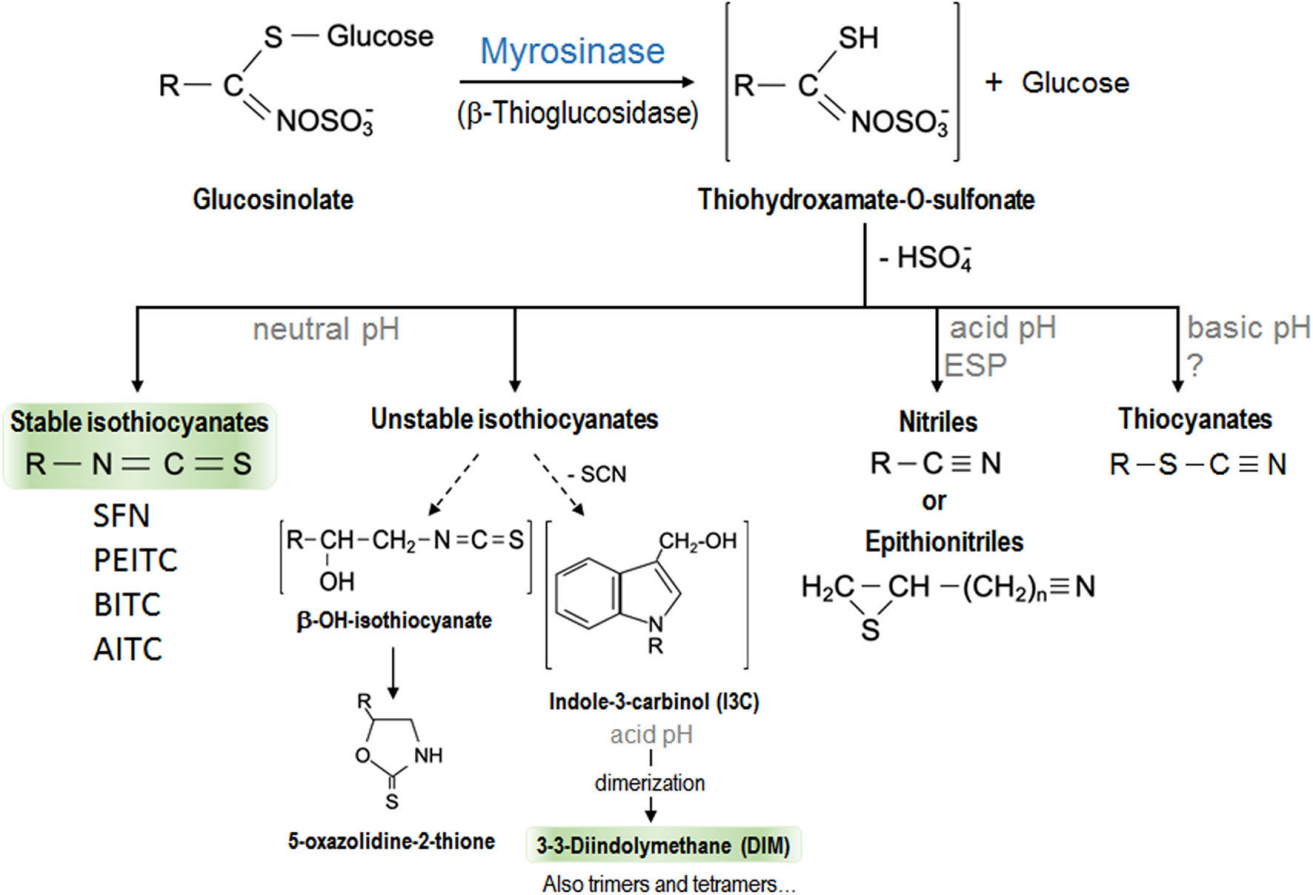
Scheme of the hydrolysis of glucosinolates by myrosinase enzyme with the resulted isothiocyanates and other derivatives. The biologically active hydrolysis products of glucosinolates, better studied and discussed in this review have been highlighted. EPS, epithiospecifer protein.

The consumption of specific cruciferous vegetables is more strongly associated with protection against cancer than that of other vegetables in general (10). The beneficial properties of vegetables are attributed to their bioactive components such as fiber, antioxidant vitamins (vitamin C and β-carotene), antioxidant minerals (selenium), and phenolic compounds. Cruciferous vegetables contain all these molecules. However, what makes them different is that they also contain some sulfur compounds called GLSs (11). Cruciferous vegetables are members of the *Brassicaceae* or *Cruciferae* family (the alternative name is due to the shape of their flowers whose four petals resemble a cross) consumed commonly, such as broccoli, cauliflower, cabbage, kale, Brussels sprouts, Chinese cabbage, radish, wasabi, mustard, and watercress. GLSs are responsible for their characteristic pungent odor and bitter taste. More than 120 different GLSs have been identified from several plants with a profile and quantity that vary depending on the cultivars and growing conditions, since these compounds are vital for plants' defense against biotic and abiotic stress (12–14). All GLSs share a basic chemical structure that contains a β-D-thioglucose group, a sulfonate oxime group, and a side chain derived from a branched chain amino acid, methionine, alanine, phenylalanine, tyrosine, or tryptophan (14, 15). According to their structure, they can be classified into aliphatic (derived from methionine, alanine, leucine, isoleucine, or valine), aromatic (derived from phenylalanine or tyrosine), and indolic (derived from tryptophan) GLSs (16). GLSs are biologically inactive and need to be hydrolyzed by the enzyme myrosinase to become active (12). GLSs and myrosinase are located at different compartments in intact plant cells. When the structure is damaged, both molecules come into contact and the reaction occurs. This usually occurs when the vegetables are cut or damaged (during harvesting and processing) or during chewing (17). Myrosinase removes β-D-thioglucose from GLS, leading to the formation of unstable compounds that finally become bioactive molecules such as thiocyanates, ITCs, indoles, and nitriles in a process influenced by the pH. At neutral pH, ITC formation is favored, while an acidic pH favors those of nitriles (13, 18) which do not have anticarcinogenic properties. In addition, in some plants and specific GLSs, specific proteins (different from myrosinase) could also play a role in these chemical changes. These include epithiospecifier protein (ESP) which drives the reaction of nitrile formation in plants that contain alkenyl-GLSs but not those that contain GLSs with a terminal alkene (13). Figure 1 shows a scheme of GLSs derivatives formation. The amount of GLSs ingested, and their active compounds (the ITCs) which can eventually reach the tissues, is determined by several factors including the content of GLSs determined by the variety of cruciferous vegetables consumed, the lability of GLSs to environmental conditions and cooking techniques, their bioavailability, and their metabolism (19). Table 1 contains a summary of the most abundant cruciferous GSLs, their derivative ITCs/indoles, and plants in which they are found.

**Table 1:** The most abundant and studied GSLs, their ITCs derivatives and representative cruciferous-where found.

**Glucosinolate**	**Isothiocyanate (abbreviation)**	**Cruciferous plant**
Glucobrassicin	Indole-3-carbinol (I3C)	All cruciferous
Sinigrin	Allyl isothiocyanate (AITC)	Mustard Brussels sprouts Cauliflower
Glucoraphanin	Sulforaphane (SFN)	Broccoli Arugula
Gluconasturtiin	Phenyl isothiocyanate (PEITC)	Cabbage Chinese cabbage Radish Watercress
Glucotropaeolin	Benzyl isothiocyanate (BITC)	Garden cress Horseradish White mustards

## Abundance, Lability, Bioavailability, and Metabolism of GLSs

The content of GLSs varies widely depending on the cruciferous variety and is conditioned by the place and conditions of cultivation (12). Thus, it has been reported that cultivation under low temperatures decreased plant GLS levels; thus, plants harvested in the winter or autumn contain lower GLS levels than those harvested in the spring or summer (20, 21). In addition, the post-harvest and packaging conditions also modify the concentrations of GLSs in cruciferous vegetables (22). Rodrigues et al. (23) found a 79% reduction in the total GLS concentration in freshly harvested broccoli inflorescences stored at room temperature for 5 days, while those refrigerated at 4°C showed a 16% decrease. Therefore, a refrigerated mode of transport as well as the sale period could imply a significant loss of these compounds, which emphasizes the importance of consuming local vegetables. On the other hand, the cooking technique utilized may also cause more or less leakage of GLSs; however, the data about this are controversial as many variables need to be taken into account, such as whether the vegetable is chopped, the amount of water, cooking temperature, and duration of cooking (22, 24–26). Data emanating from diverse studies show that boiling leads to the largest loss of GLSs (by >50%), depending on the time and volume of water more than the temperature. During boiling, GLSs are mostly lost through leaching into the cooking water, and is favored by the convection movements of boiling water (27). The data led to the conclusion that boiling with greater amounts of water and for longer periods increases the losses of GLSs. Microwave cooking also leads to loss of GLSs although the increased osmotic pressure causes a breakdown of the cell structure which brings the GLSs and myrosinase into contact. The losses described are very variable (15–75%) and depend on factors such as the amount of water, the intensity of microwave radiation, and the duration of cooking (22, 25). Stir frying leads to an increased loss of GLSs (by >60%) (25, 28). In this case, temperature is the principal feature. It was observed that stir fried broccoli cooked for 3–5 min with oil preheated at 200°C did not lead to a significant change in the GLS content (27). However, cooking in preheated oil and maintenance at 140°C for 5 min led to losses of about 60% (25). Lastly, data showed that steaming was the best mode of cooking that ensured the preservation of GLSs. Most studies found little or no effect (26, 28), and some studies reported that an increase in GLS content (24, 28) was favored perhaps because steaming strongly limits their leach into the water.

There is evidence that very little of intact GLSs are absorbed (29–31) and it is their hydrolyzed products (the ITCs and indoles) that are absorbed (31). Chopping and chewing breaks the plant structure and promotes the hydrolyzation of GLSs to ITCs by myrosinase; however, the cooking temperature can inhibit the activity of myrosinase and limit the formation of ITCs (22). Therefore, it has been found that after ingestion of cruciferous vegetables, the myrosinase from gut microbiota has relevant influence in the conversion of inert GLSs to bioactive ITCs (32–36). Consequently, intact GLSs may reach the large intestine where they can be degraded by the resident gut microbiota leading to the release of ITCs which will then be absorbed (31, 32, 37, 38). Recently, it has been found that the consumption of broccoli modifies the gastrointestinal microbiota to a healthier profile (39), which coincides with that of bacterial genera that have myrosinase activity (33–35). Finally, ITCs must be absorbed and distributed by the body to reach the tissues where they will affect their biological mechanisms. The precise mechanism by which ITCs are absorbed has not been elucidated completely; however, numerous studies conducted using human (37, 40–42) and animal models (43–46) have described the mechanism through which ITCs are absorbed. Most studies conducted among humans have assessed absorption indirectly through the determination of their metabolites in urine or by the cyclo-condensation method (37), or by high performance liquid chromatography with tandem mass spectrometry (HPLC MS/MS) to measure plasma ITCs (42) after an oral dose. In studies conducted among humans after intake of a single dose of fresh broccoli, the peak plasma concentration of ITCs (mainly sulforaphane [SFN]) was found at about 3 h and disappeared at 24 h while the peak concentration in urine was recorded around 6 h and disappeared at 24 h (41, 42). Among rats, studies mostly assessed the fate of an oral dose of ^14^C-labeled ITCs. In general, rapid absorption of ITCs was observed but with some differences between individual compounds as the structure may affect the liposolubility (46, 47). For example, among animals given a ^14^C-phenethyl ITC (PEITC), the radioactivity in plasma peaked at 2.9 h while among those who received ^14^C-PHITC (phenylhexyl ITC), the peak plasma level was recorded at 8.9 h. Nevertheless, those that received ^14^C-PHITC excreted only about 7% in urine and 47% in feces in contrast to those that received ^14^C-PEITC who eliminated the majority in urine (about 89%) and only 10% was recovered in feces. These different absorption rates could perhaps be attributed to a greater lipophilicity of PHITC (46). All fractions of ITCs—those that are already present in previously consumed food, those that are formed during chewing, and those that result from microbiota activity—are absorbed by the epithelial cells of the small intestine or colon (31, 48). Available data indicate that the entry of ITCs into enterocytes through the apical membrane by passive diffusion, which is facilitated by rapid conjugation with reduced glutathione (GSH) by means of the enzyme glutathione-S-transferase (GST), results in the maintenance of the concentration gradient and favors rapid internal accumulation of GSH-ITCs (49, 50). GSH-ITCs are released through the basolateral membrane via multidrug-resistance-associated protein 1 (MRP1) and P-glycoprotein (P-gp) as occurs in other cell lines (51). In addition, some authors revealed that a proportion of absorbed ITCs and GSH conjugates (GSH-ITCs) effluxed back into the lumen as GSH-ITCs (49). Once absorbed, peak plasma concentrations of GSH-ITCs decline rapidly, signifying a rapid distribution (37, 52, 53). In blood, GSH-ITCs maintain an equilibrium with free ITCs due to the low concentrations of GSH in plasma and they are taken up by tissue through a similar mechanism within the enterocytes, passive diffusion, and formation of GSH-ITCs (49–51, 54). The capacity to synthesize GSH-ITCs is determined by the intracellular concentration of GSH and GST activity and could be related to the specific effects of ITCs in different tissues (31). Kim et al. confirmed that GSH-AITC accumulated to a greater extent in the liver within the first hour after an oral dose of AITC (25 mg/kg of body weight) followed by the kidneys, spleen, lungs, and heart (53). On the other hand, unstable ITC derivatives such as I3C (the major product of hydrolysis of indole-GLSs) in the acidic pH of the stomach are converted into condensed products (mainly, DIM [diindolylmethane] which is the most active) (55). De Kruif et al. in rats given 13C orally found DIM in tissues extracts from the stomach, small intestine and liver, proving that they are absolved by the small intestine (56). Anderton et al. determined the tissue distribution of DIM in mice after an oral load of I3C, and found that the liver retained higher levels (129 μg/g) of DIM, followed by the lungs (70.5 μg/g), kidneys (61.8 μg/g), heart (54.2 μg/g), and brain and plasma (19 μg/g). In addition, the peak plasma concentration of DIM occurred at 2 h after I3C administration (52, 57). In human studies, following I3C administration, the peak plasma concentration of DIM was detected after ~3 h (58); the lymphocyte activity of GST had increased (59) and urinary DIM was detected after ingestion of brussels sprouts (60).

**Figure 2:**
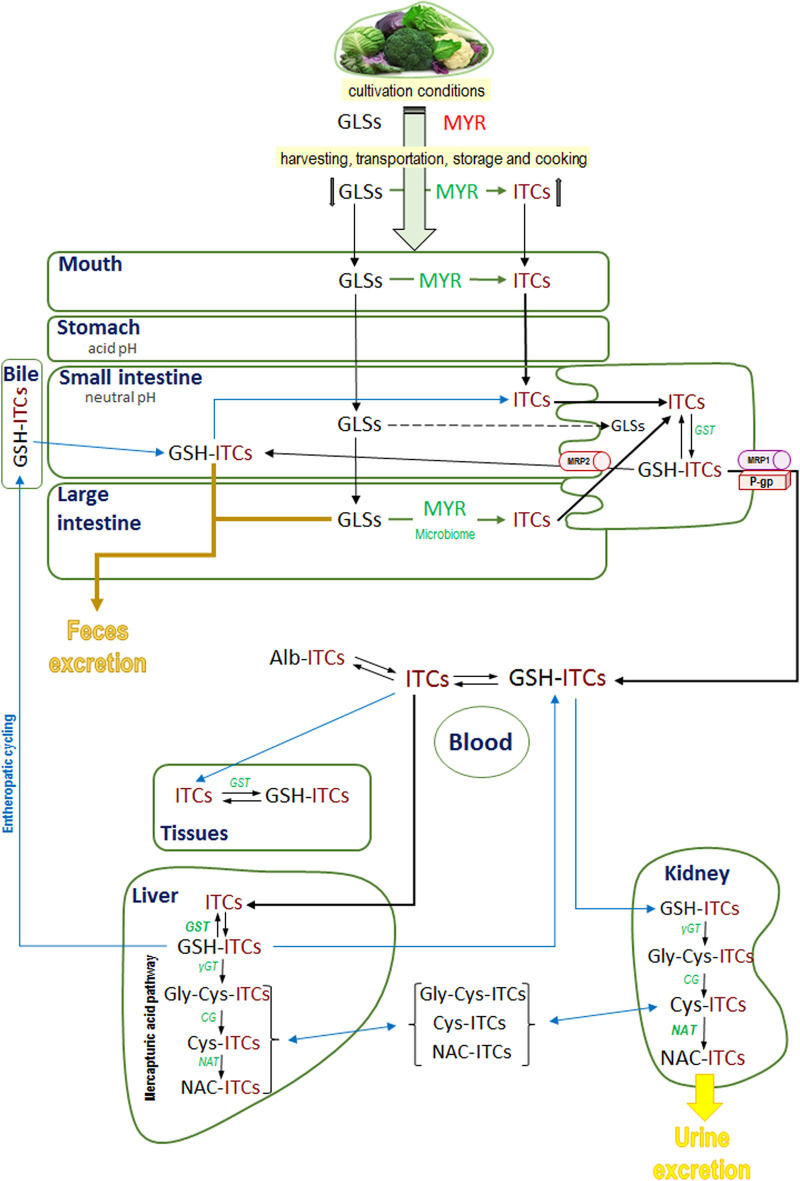
General overview of ingested glucosinolates and isothiocyanates fate: mouth hydrolysis, microbiota hydrolysis, absorption, metabolism, and excretion. GLSs, glucosinolates; ITCs, isothiocyanates; MYR, myrosinase; GSH-ITCs, glutathione isothiocyanates conjugates; Alb-ITCs, albumin isothiocyanates conjugates; Gly-Cys-ITCs, glycine-cysteine isothiocyanates conjugates; Cys-ITCs, cysteine isothiocyanates conjugates; NAC-ITC, N-acetylcysteine isothiocyanates conjugates; GST, glutathione-S-transferase; γGT, γglutamyltranspeptidase; CG, cysteinylglycinase; NAT, N-acetyltransferase.

After the initial conjugation with GSH catalyzed by the enzyme GST, the conjugates are metabolized to a mercapturic acid derivate (N-acetyl-cysteine-ITC [NAC-ITC]), which is excreted in the urine (53). Thus, the liver plays a relevant role in xenobiotic detoxification since it contains high levels of GSH and has the highest GST activity in the organism, and supports the formation and accumulation of high levels of GSH-ITC conjugates (52, 53). On the other hand, the kidney is the major organ implicated in the conversion of GSH-ITCs to the corresponding mercapturic acid derivate since it has elevated N-acetyltransferase activity (NAT) (61). Thus, it appears from experimental data (62) that the GSH-ITCs initially formed in the liver would be secreted as such and those formed in the kidney would be acetylated and excreted. In support of this, Kim et al. found that the level of GSH-AITC was higher in the liver but the level of NAC-AITC was more elevated in the kidney (53). In addition, the GSH-ITCs in the liver can also be metabolized to NAC-ITC and then excreted into the bile (62). The enterohepatic cycling was supported by evidence from human data that the excretion of dihydrocarbonates (such as GSH-ITCs) showed a biphasic curve after ingestion of horseradish (29). Data show that the specific structure of ITCs could affect their affinity to the enzyme GST, act as a phase I enzyme substrate, and be secreted into the bile and excreted in feces. For example, phenyl-, benzyl-, and allyl-ITCs such as PEITC are principally conjugated with GSH and metabolized via the mercapturic acid pathway to NAC-PEITC and excreted in urine. In contrast, a majority of PHITC (with an alkyl side chain) is excreted in the bile and recuperated in feces (46, 62, 63). Thus, phase I metabolism could contribute to the biotransformation and bioavailability of active ITCs. Figure 2 shows a schematic representation of the factors influencing the amount of GLSs ingested, their absorption, distribution, metabolism, and excretion.

## Biological Effects of ITCs and Mechanisms

As mentioned above, numerous epidemiological studies that reported a lower risk of suffering various types of cancers when cruciferous vegetables are consumed in the diet in a regular form have been published over the years. From these observations, abundant efforts have been made to understand the mechanisms through which hydrolyzed products derived from GLSs reduce the risk of cancer with the aim of arriving at potential cancer therapies. The most studied products of hydrolysis of GLSs, due to the abundance of their precursors in consumed cruciferous vegetables, have been the stable ITCs PEITC, SFN, BITC, AITC, and the unstable ITC derivatives (the indoles I3C/DIM). This section of the review focuses on the biological effects of ITCs and indoles that have been described in the regulation of key cell signaling pathways. These effects have been firstly and widely studied in relation to cancer. However, these pathways are not only altered in cancer but also in other conditions such as obesity, diabetes, and arteriosclerosis (MetS). This is why most of the cited studies will refer to cancer (though not all) and we reserve the final section of the review to focus on how ITCs and indoles can prevent MetS by modulating the common pathways described in cancer. It has been found that ITCs and indoles modulate the activity of enzymes involved in phases I and II of the detoxification pathway; modulate the cell cycle, oxidative stress, and inflammation; angiogenesis; as well as epigenetic effects.

### Modulation of Enzyme Activity in Phases I and II of the Detoxification Pathway

Phases I and II of the detoxification pathway contain the most important enzyme group involved in xenobiotic biotransformation (64). The phase I enzymes are represented by the cytochrome P450 family (CYP450) that modify the molecule via oxidation, reduction, or hydration and, as a result, the xenobiotic (e.g., drugs, procarcinogens) may be activated or inactivated. The phase I metabolism generally increases the polarity of molecules and facilitates the excretion. Phase I reactions can be followed by phase II reactions to increase solubility, but phase I reactions are not a requirement to be substrate of Phase II enzymes. Phase II consists of a sulfation, glucuronidation, acylation, methylation or conjugation with GSH or amino acids, facilitating its excretion in urine or bile (64). However, some potential carcinogenic molecules become active when they are biotransformed through phase I enzymes (65). Inhibition of specific CYP enzymes involved in carcinogen activation inhibited cancer development in animal models (65, 66).

Stable ITCs (such as SFN, AITC, PEITC, and BITC), have been found to inhibit carcinogen activation through the inhibition of CYP enzymes in animal studies and cell lines (67–70). Leclercq et al. found that in human volunteers, a single ingestion of watercress homogenate (rich in the GLS precursor of PEITC) increased the area under the curve of time course plasma chlorzoxazone, suggesting that CYP2E1 activity was inhibited (71). Yoshigae et al. finally described a possible mechanism underlying the inactivation of human CYP2E1 by PEITC (72). Later, Nakajima et al., in an *in vitro* model of cells expressing specific human CYP isoforms, proved that PEITC yielded chemopreventive effects against nitrosamine-induced carcinogenesis through CYP450 inhibition (67). On the other hand, indole-GLS derivatives (such as I3C) and their condensed products (such as DIM) have a different effect than stable ITCs since they increase the activity of certain CYP enzymes (68, 69, 73). The mechanism described would be that DIM binds to the aryl hydrocarbon receptor, which then recognizes and binds to the xenobiotic response element (XRE) sequences in the DNA of genes of a large number of CYP enzymes, and increases their expression (74, 75). Increased expression of phase I enzymes in general could be considered beneficial to the elimination of the possible adverse effects of activated procarcinogenic agents. In breast cancer (characterized by altered CYP expression) I3C/DIM demonstrated a protective effect (73). In some breast cancer cell lines, CYP1A1 and CY1A2 are expressed at low levels; they are responsible for the production of estrogen metabolites that are protective against cancer, and it has been found that DIM increases their expression, favoring a protective role (76). Therefore, in a phase I clinical trial of healthy women, it was observed that the administration of I3C promotes CYP1A2 activity (59).

**Figure 3:**
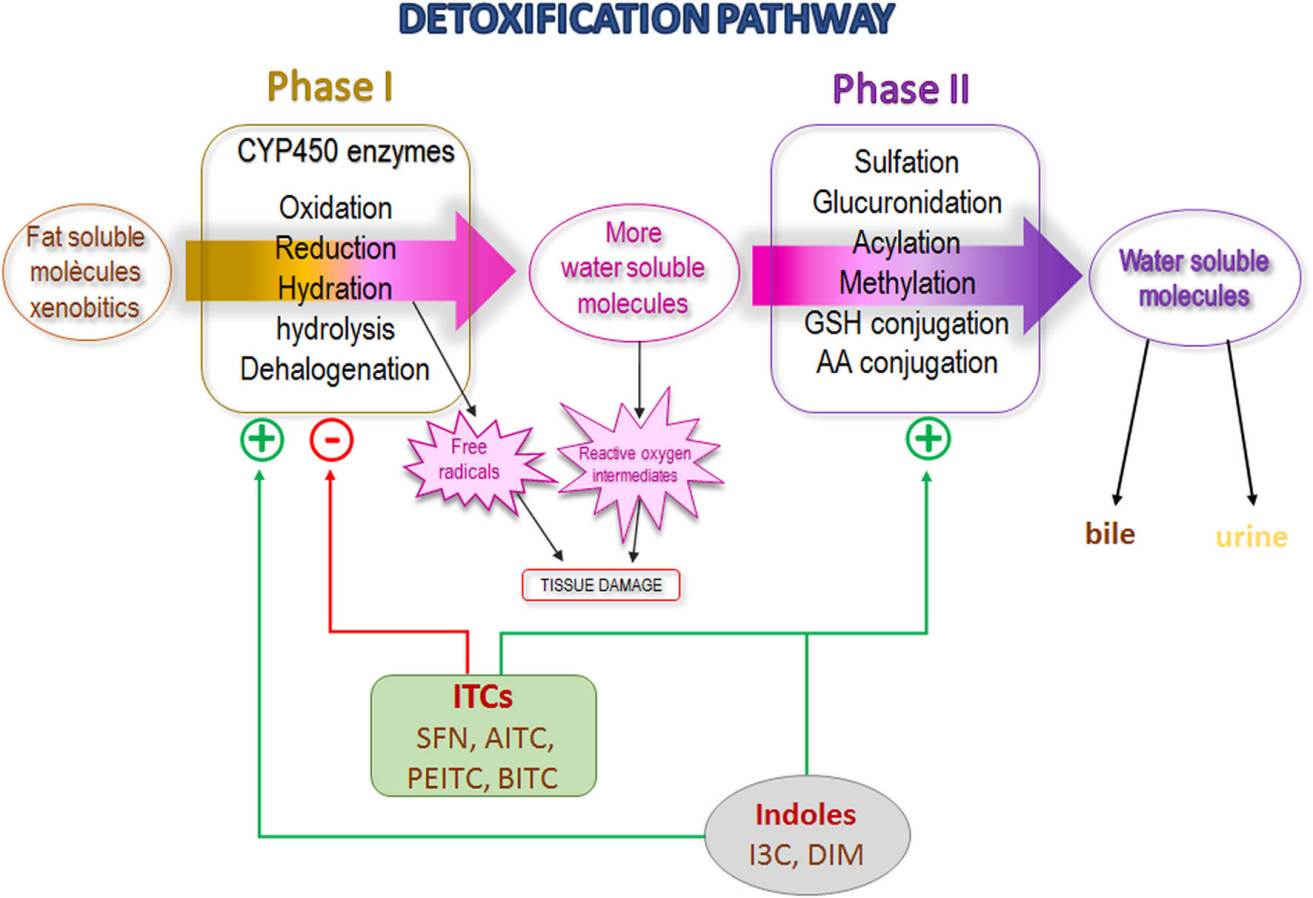
Detoxification pathway scheme showing isothiocyanates and indoles effect. SFN, Sulforaphane; AITC, Allyl isothiocyanate; PEITC, Phenyl isothiocyanate; BITC, Benzyl isothiocyanate; I3C, Indole-3-carbinol; DIM, diindolylmethane.

Numerous stable ITCs and indole derivatives of GLSs are potent inducers of phase II enzymes (49, 64, 77–79). The genes that encode phase II enzymes contain antioxidant response element (ARE) domains; ITCs recognize and bind to them, increasing their expression (68). A study conducted among smokers showed that watercress consumption promotes a higher excretion of glucuronidated nicotine in urine indicating activation of the phase II enzyme UDP-glucuronosyltransferase (UGT) (80). Most recently, Yuan et al., in a randomized, placebo-controlled, double-blind study conducted among smokers found a greater excretion of the mercapturic acids of benzene and acrolein (substances of tobacco smoke) in urine after oral administration of PEITC for 5 days (81). In addition, human polymorphisms for the *GST* gene that affect their activity have been described (82). Lower or null GST activity could affect the susceptibility to diseases due to reduced excretion of toxic molecules or could promote increased efficiency of cancer chemotherapy owing to a higher drug half-life (83). In the same sense, lower or null GST activity could result in a slower elimination and longer exposure to ITCs in cruciferous vegetables, maintaining their biological actions for a longer time. Several epidemiological studies found a higher cancer protective effect of cruciferous vegetable consumption among individuals carrying *GSTM1-null* and *GSTT1-null* than among those with normal GST activity (84–87). In the same vein, Yuan et al. (81) determined urine excretion of mercapturic acids of benzene and acrolein in cigarette smokers who received oral supplementation of PEITC, and observed a remarkably stronger effect of PEITC in subjects with *GSTM1* and *GSTT1-*null genotype with a significant higher excretion than in those with a normal genotype (88). In Hep G2-C8 cells, Saw et al. described that I3C/DIM induced the expression of ARE-mediated phase II genes such as *GSTM2, UGT1A1*, and *NQO1(NAD(P)H:quinone oxidoreductase 1*, as well as a synergic effect with SFN and PEITC (89). In summary, the main mechanism of carcinogenesis inhibition described for ITCs appears to occur through two levels of the detoxification pathway: selective P450 enzyme inhibition (stable ITCs) or activation (I3C/DIM) and an induction of phase II enzymes, preventing the activation of procarcinogens and increasing their excretion (Figure 3). Although these activities are attributed exclusively to ITCs, an *in vitro* study described that intact GLSs modulate hepatic CYP450 and phase II enzymes (78).

### Modulation of the Cell Cycle: Proliferation and Apoptosis

The cell cycle comprises a series of tightly regulated stages that include growth and division to eventually result in two new daughter cells. Multiple checkpoints are implicated in the regulation of the cell cycle such as growth signals, availability of nutrients, and the integrity of DNA. If the DNA is damaged, the cell cycle can be transiently arrested to repair it or the apoptosis pathway can be activated, leading to cell death. When cell cycle regulation is disturbed, the DNA is not repaired correctly and the mutation can propagate, contributing to the development of cancer (19). The effects of ITCs/I3C on cell cycle regulation have been studied in a wide variety of cancer cell lines and in animal models (55, 69, 90, 91). Numerous *in vitro* experiments in an extensive variety of cancer cell line models of the bladder, lung, prostate, osteosarcoma, adenocarcinoma, and colon (UM-UC3, J82, RT4, LTEP-A2, PC-3, LM8, and HT-29) have investigated the effects of SFN on cell cycle regulation. The results showed an extensive coincidence in that SFN arrests the cell cycle in the G2/M phase (92–100). Some of these studies also showed that its effect was mediated through induction of the expression of p21 and p53, which are cyclin-dependent kinase (CDK) inhibitors (94–96). Cyclins and CDK are important promoters of mitosis initiation as a consequence of cellular proliferation. In a manner similar to SFN, PEITC arrests the cell cycle in the G2/M phase in HeLa, Caco-2, and PC-3 cell lines (101–103), but in the G0/G1 phase in human prostate cancer DU-14 and LNCaP cell lines (104). In most of these studies, an increase in expression of p21 and a decrease in expression of the cyclins CDK and cdc25c was found (102, 103). Other studies regarding the effects of BITC and AITC on cell proliferation also showed their ability to arrest the cell cycle generally in G2/M by increasing p21 and/or p53 expression and decreasing the expressions of CDK and cyclins (cdk1, cyclin B) and cdc factors (cdc2, cdc25b, cdc25c) (93, 101, 105–111). The effects on cell proliferation have also been described for I3C/DIM; in MCF7 breast cancer cell lines, they arrest the cell cycle in G1 (112–114). Moreover, I3C/DIM increase the expressions of p53 (114), p21, and p27 and decrease that of CDK6 (112). Similar effects were observed in PC-3 prostate cancer cells treated with I3C (115).

In addition, the mechanisms through which ITCs inhibit the proliferation of cancer cells include the activation of apoptosis, as well as both the mitochondrial/intrinsic pathway and death receptor/extrinsic pathway (91). All the most studied ITCs (such as PEITC, SNF, BITC, AITC, and I3C/DIM) have been found to cause inhibition of the antiapoptotic factor Bcl-2 in different cell lines and mice (93, 94, 110, 116, 117). However, different mechanisms through which ITCs activate apoptosis have been described. Hu et al. (118) described that PEITC induces apoptosis via the intrinsic pathway through MAPK signaling, via activation of JNK through p38 that leads to cytochrome c release, and subsequent activation of caspase-9 and caspase-3 in HT-29 cells. In another study, Wang et al. (119) showed that induction of apoptosis by PEITC in PC-3 cells is mediated by ERK. Later, Xu et al. (120) demonstrated that ITC-induced apoptosis (by SFN, PEITC, and AITC) is tightly coupled to ERK and JNK signaling in human prostate PC-3 cells. Furthermore, in the bladder cancer cell lines HTB9 and RT112, Islam et al. (121) found that SFN inhibits proliferation of cells by decreasing the expressions of PI3K/Akt/mTOR. Other studies of 5637 and T24 human bladder cancer cells showed that treatment with SFN induces mitochondrial caspase dependent apoptosis due to reactive oxygen species (ROS) accumulation and activation of endoplasmic reticulum stress with activation of Nrf2 (nuclear erythroid 2-related factor 2) signaling pathway (121, 122). Other researchers have confirmed that the increase in ROS levels is a way by which BITC and PEITC induce apoptosis in cell lines of pancreatic cancer, chronic myeloid leukemia, and chronic lymphocytic leukemia (123–125). Nrf2 is a transcriptional factor that binds to ARE regions in DNA to induce the expression of major antioxidant enzymes that detoxify ROS (126, 127) and can inhibit the nuclear factor kappa B (NF-κB) transcriptional factor. NF-κB activity is linked to cellular processes that promote cancer, such as inflammation, cell proliferation, and angiogenesis and facilitate tumor growth and metastasis (128, 129); thus, its inhibition could yield positive effects in the prevention or treatment of cancer. The evidences that SFN and BITC decrease NF-κB activity have been described in the bladder and colon cancer cell lines T24 and T29, respectively (130, 131). Chen et al. showed that DIM also promotes apoptosis through AMPK activation, resulting in suppression of mTOR in C4-2B and LNCaP prostate cancer cells (132). In addition, in PC-3 prostate cancer cells, it was found that I3C/DIM induces apoptosis through inhibition of Akt/PI3K signaling with decreases in the expressions of Bcl-xL and BAD (133). Thus, I3C/DIM induce apoptosis via modulation of the PI3K/Akt/mTOR/NF-κB pathway (115, 134). Finally, it has been reported that ITCs and indoles play an important role in controlling the cell cycle, diminishing the proliferative capacity, and activating the apoptosis pathway; however, the specific underlying mechanism could differ according to the ITC or cell type.

### Antioxidant and Anti-inflammatory Effects

Usually, oxidative stress is accompanied by an inflammatory response that is part of the responses to combat this oxidative damage. However, persistence of an oxidative stimulus (even if mild) will also lead to the maintenance of a chronic inflammatory state that can be more or less severe. Therefore, the organism fights this oxidative damage first to eliminate the causative agent and then to eliminate the inflammation to restore normalcy (135). As mentioned above, the genes that encode proteins relevant to the elimination of oxidative agents contain ARE sequences, as do phase II enzymes and stress-responsive antioxidant genes, such as *GST, UGT, NAD(P)H:quinone oxidoreductase 1* (*NQO1*) and *heme oxygenase-1* (*HO-1*) (136, 137). The central role of Nrf2 transcription factor in the upregulation of antioxidant genes in the oxidative stress response has been described (138). NF-κB plays a key role in immune response regulation to activate mediators (such as pro-inflammatory cytokines) to favor the attack of the inciting agent in the first step of the immune response, in which it also produces oxidation agents (135). However, in most normal cells NF-κB is in inactive form, and a constitutive activation of NF-κB has been noted in almost all cancers (128) and chronic physiological inflammatory conditions such as MetS (139). It has been reported that ITCs and indoles increase Nrf2 activity and inhibit NF-κB (140–145). In Raw 264.7 macrophage cells, it was found that BITC diminished the lipopolysaccharide (LPS) induced inflammatory response in a dose-dependent manner through the inhibition of NF-κB translocation to the nucleus as well as that of its target genes *Il*β*1, IL6, TNF*α, nitric oxide synthase (*iNOS*), and cyclooxygenase-2 (*COX2*) (145). In mice, topical application of BITC also ameliorated the TPA-induced swelling in the ears (model for inflammation diseases, including psoriasis) diminishing the expressions of *iNOS* and *COX2* (145). The authors concluded that the anti-inflammatory effects of BITC could be mediated via the inhibition of Akt and ERK and the subsequent downregulation of NF-κB signaling. Zhou et al. recently confirmed that AITC attenuated oxidative stress in mice with chronic obstructive pulmonary disease via Nrf2 signaling (140). In a traumatic brain injury model of mice, AITC reduced infarct volume and brain swelling by decreasing the levels of pro-inflammatory molecules, IL1β, IL6 through the downregulation of NF-κB, and ameliorated some neuronal plasticity markers and increased the antioxidative mechanism by upregulating the Nrf2 pathway, evidenced by the double effect of AITC (141). Similar results have been described for PEITC, BITC, and SNF, in that they reduce the endothelial damage induced by oxidized LDL (146) or in NIH3T3 fibroblasts (142). Kim et al. described the protective effect against inflammation induced by DIM, but not for I3C, in LPS-treated BV-2 microglial cells by decreasing the expressions of iNOS, COX2, and NF-κB (147). In addition, DIM reduces the hippocampus inflammation caused by LPS administration in mice, thereby reducing the infiltration of pro-inflammatory macrophages (147). In mice, it has been found that DIM also attenuates colonic inflammation and tumorigenicity in a mouse colitis model and an azoxymethane (AOM)/DSS induced colon cancer model (148).

### Anti-angiogenic Effects

In the stages of cancer development, progression refers to the capacity for tumors to extend to other parts of the body (also referred to as metastasis). Angiogenesis denotes the construction of new blood vessels from existing ones to facilitate the delivery of nutrients and oxygen to tumor cells. Therefore, angiogenesis plays a fundamental role in tumor progression, and thus metastasis, which constitutes the most difficult phase for cancer control and treatment and is normally the cause of death (149–151). Metastasis is a complex process whereby cells lose their adhesive capacity and migrate through the circulation to other tissues. Thus, the new vascular network facilitates migration of cells; however, extracellular matrix degradation is an essential aspect of the loss of cell adhesion capacity. In this process, enzymes called metalloproteinases (MMPs) are important. There are pieces of evidence that SNF, PEITC, BITC, AITC, PITC, and I3C downregulate MMP2 and MMP9 in various cell lines (152–154). The angiogenic process is regulated by numerous molecules, in addition to MMPs, such as growth factors (vascular endothelial growth factor [VEGF], bFGF, angiopoietins, EGF, and TGF), integrins, and interleukins (IL1, IL2, IL6, IL8, IL12, and IL17) (149). The most studied among them is VEGF and there is evidence that ITCs and I3C inhibit angiogenesis by downregulating VEGF (152, 155). Treatment of HUVEC and PC3 cells with PEITC inhibited neovascularization and cell migration with suppression of VEGF secretion, downregulation of VEGF receptor 2 protein levels, and inactivation of Akt (156). Treatment of human microvascular endothelial cells (HMEC-1) with SFN decreases the formation of new microcapillaries with reduction in VEGF levels (152). This was also observed later by Kim et al. in HCT116 human colon cancer cells (157). Thejass et al. showed that AITC and PEITC treatment downregulated VEGF and pro-inflammatory IL1β, IL6, and TNFα, but increased the anti-inflammatory effects of IL2 and inhibition of metalloproteinase (TIMP1) (158). Among mice, Hajra et al. reported that I3C blocked angiogenesis by inhibiting VEGF-A and MMP-9 (155). One important factor that stimulates angiogenesis is hypoxia that results from tumor growth or due to other situations such as increase in adipose tissue caused by obesity, leading to the activation of hypoxia inducible factor-1α (HIF-1α) which in turn stimulates VEGF (152). In accordance with this, it was found that SFN dramatically decreases the expressions of HIF-1α and VEGF in HCT116 cells exposed to hypoxia (157). Similar results were found in MCF-7 cancer cells treated with PEITC (159, 160). PC-3 and C4-2B prostate cancer cells treated with DIM demonstrated decreases in HIF-1α and NF-κB expression and an increase in radiation efficacy (161). Gupta et al. described that in human glioma cells the inhibitory effect of PEITC on angiogenesis depended on PI3K/Akt and ERK/MAPK (160). In addition, it is known that IL1β upregulates HIF-1α through a classical inflammatory signaling pathway involving NF-κB and COX-2, ending in upregulation of VEGF. Thus, HIF-1α is recognized as a pivotal transcription factor linking the inflammatory and oncogenic pathways (127). The inhibition of NF-κB appears to be important in reducing cell proliferation, inflammatory status, and metastasis, and as discussed previously, there is abundant evidence that ITCs/indoles downregulate NF-κB activity (144).

### Epigenetic Effect: Histones Modification, DNA Methylation, and miRNA

The main mechanisms underlying epigenetic processes are post-translational histone modifications and DNA methylation, which are modulated by histone deacetylases (HDACs) and DNMTs (methyltranspherases), respectively. Histone acetylation results in an open chromatin conformation which facilitates the transcription process. HDACs remove the acetyl group from histones, leading to chromatin condensation which hinders transcription. The overexpression of HDACs are a common hallmark of cancer and their inhibition is considered to be therapeutic against cancer tumor progression (162, 163). ITCs and indoles have been found to exert a potent effect as HDAC inhibitors in various cancer lines (164–174). The most studied is SFN and the most common effect found is the inhibition of HDAC3 associated with an increase in p21and Bax expression and a decrease in cyclin D1 expression, which together results in cell cycle arrest and apoptosis activation (167, 168, 171, 173). In addition, in TPA-induced neoplastic transformation of mouse skin cells, Su et al. described that SFN promotes a decrease in HDAC expression with an increase in Nrf2 expression and their downstream target genes (such as *HO-1* and *NQO1*) of phase II enzymes (169). PEITC in LNCaP prostate cancer cells inhibits HDAC3 with an increase in p21and p27 expression, leading to cell arrest (170). In another experiment, the inhibition of HDAC1, 2, 4, and 6 protein expression was observed and related to activation of the tumor suppressor factor RASSF1A (Ras-association domain family 1 isoform A) (175). In addition, Beaver et al. showed that DIM significantly decreased HDAC activity and was correlated with increased expression of p21 in PC-3 and LNCaP prostate cancer cells (166). In human pancreatic cancer cells, Sanjay et al. showed that BITC incubation promotes a decrease in the expression and activity of HDAC1 and HDAC3 with decreases in the expression and activity of cyclin D1 and NF-κB (172). In accordance, I3C and DIM decrease the expression of HDAC1, 2, 3, and 8 in T cells activated by staphylococcal enterotoxin B with a decreased secretion of pro-inflammatory cytokines (174). DNA methylation is an important process by which gene transcription is regulated (176). Changes in the pattern of methylation of genes have been related to the expression of oncogenes and cancer development (176). It has also been described that ITCs and indoles regulate DNMTs and mediate the process of carcinogenesis (175, 177–184). SFN is the ITC that has been studied the most, and in general, the research shows that SFN promotes a decrease in the DNA methylation of certain genes with anti-tumor action through the inhibition of DNMT1, DNMT3A, and DNMT3B. This leads to decreased expression of HDACs resulting in reactivation of genes [such as *p21* (177), *cyclin D2* (179) or *Nrf2*] and the downregulation of HO-1 and NQO1 (169, 181) depending on the nature of the study and the type of cell utilized. Similar results were obtained in DIM-treated TRAMP-C1 cells assessed by Wu et al., in which DNMT expression diminished and the methylation status of Nrf2 was reversed, resulting in its enhanced expression (184). Wong et al. described that in normal prostate cell (PeRC) and cancer cell lines (LNCaP and PC3), SFN and DIM altered promoter methylation in distinct sets of genes with similar targets within a single cell line. Further, they showed that SFN and DIM reversed many of the cancer-associated methylation alterations (178). PEITC in LNCaP cell lines also showed a reduction in the activity of RASSF1A promoter methylation through inhibition of DNMT3A and 3B (175). In leukemia T cells, the effects of PHITC were an inhibition of DNMT1 and DNMT3B, an increase in acetylation of histones 3 and 4, reversal of hypermethylation of the tumor suppressor gene *p15*, and reactivation of its transcription (180).

**Figure 4:**
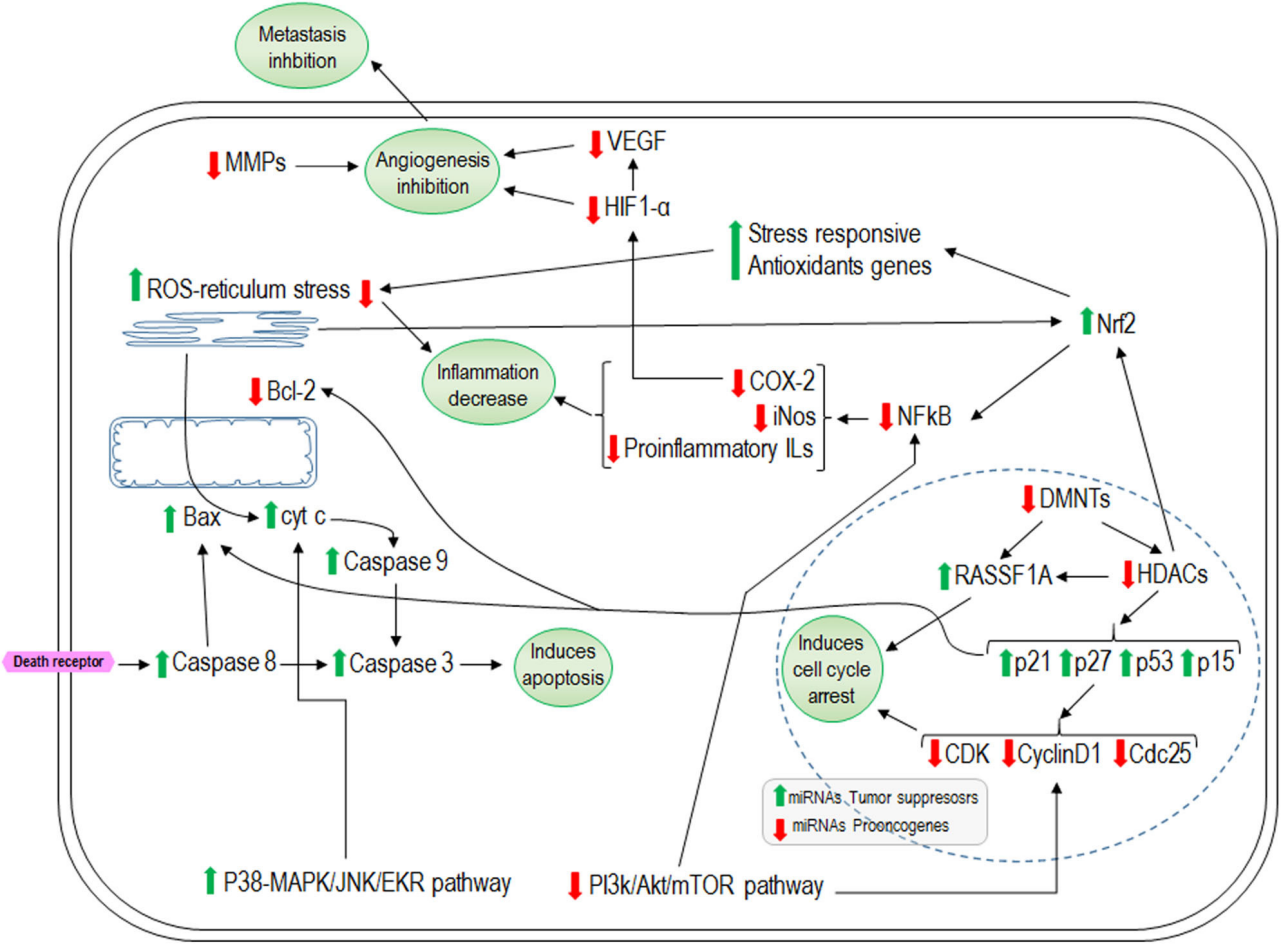
Representative effects of isothiocyanates on cellular cycle arrest, apoptosis, inflammation, and angiogenesis. Green arrows indicate the pathways that isothiocyanates increase and the red arrows the inhibited ones. HDACs, histone deacetylases; DNMTs, methyltranspherases MMPs; RASSF1A, Ras-association domain family 1 isoform A; ROS, reactive oxygen species; COX-2, ciclooxigenasa 2; HIF-1α, hypoxia inducible factor-1α; VEGF, Vascular Endothelial Growth Factor.

miRNAs are non-coding RNAs that are most abundant in animal cells. They have a length of 20–24 nucleotides and they play a pivotal role in regulatory pathways including differentiation, proliferation, and apoptosis, and regulate approximately one third of human genes (185, 186). During carcinogenesis, various miRNAs are deregulated (186). There is evidence that ITCs and indoles exert some of their effects by modulating specific miRNAs (187). It has been found that SFN in epithelial cancer cells (NCM460 and NC356) modulated 18 miRNAs (15 upregulated and 3 downregulated). Among the upregulated genes were *miR-23b* and *miR-27b* which are tumor suppressors, while the pro-oncogenic *miR-155* was downregulated (188). PEITC in PC3 prostate cancer cells causes overexpression of miR-194 which decreases the expression of MMPs and hindering metastasis (189). I3C increases miR-34a expression in MCF-7 human breast cancer cells using *p53* as a direct transcriptional target (190). In rats exposed to environmental cigarette smoke, I3C and PEITC showed a synergic effect by restoring the lung miRNA profile (191). In general, there is an increase in expression of those miRNAs related to decreased angiogenesis and proliferation or increased apoptosis and a decrease in expression of those miRNA implicated in the opposite direction (187, 192–195). Figure 4 summarizes the overall effects described for ITCs and indoles in the regulation of cell proliferation, apoptosis, inflammation, and angiogenesis.

## Metabolic Syndrome: Definition

Obesity is characterized by excess body weight due to fat excess (196). The expansibility of white adipose tissue (WAT) in obesity entails deregulation between cellular proliferation and apoptosis, pro-inflammatory macrophage infiltration with increased pro-inflammatory cytokine secretion, and stimulation of angiogenesis. Obesity is frequently associated with other pathologies, mainly type 2 diabetes (insulin resistance), atherosclerosis (dyslipidemia), and hypertension. This cluster of associated pathologies is known as the MetS (197) and is currently a leading cause of death worldwide (198).

Several health organizations have provided definitions for MetS. The United Kingdom National Health Service (199) defines MetS as “… the medical term for a combination of diabetes, high blood pressure (hypertension), and obesity” and adds that “…it increases the risk of coronary heart disease, stroke, and other conditions that affect the blood vessels.” The International Diabetes Federation (IDF) (200), the American Heart Association (201) and the National Heart, Lung and Blood Institute (202) define MetS as a group of risk factors for heart attack or stroke. Specifically, the IDF posits that MetS “… is a cluster of the most dangerous heart attack risk factors: diabetes and raised fasting plasma glucose, abdominal obesity, high cholesterol, and high blood pressure.” According to data from the World Health Organization (2016), the leading cause of death worldwide was ischemic heart disease, followed by stroke and chronic obstructive pulmonary disease (198) all of which are related to MetS. Scientists have agreed on a set of criteria for the diagnosis of MetS. According to the IDF, for a person to be diagnosed with MetS, they must have: central obesity (defined according to the waist circumference with values specific to the ethnicity) (203) plus any two of the following four factors: raised triglyceride levels, reduced high density lipoprotein cholesterol level, raised blood pressure, and increased fasting plasma glucose concentration (200).

### Obesity and Comorbidities

Obesity, the condition central to MetS, is characterized by an excess body weight due to fat excess (196). Today, obesity is defined as a mild but chronic inflammatory state (204). Animal studies have shown that the sequence of obesity establishment resembles the stages of the typical inflammatory response, only that it does not resolve in this case (205–207). In mice fed a high fat diet (HFD), it has been observed that in the first week, there already exists an increased infiltration of neutrophils in WAT (206). Then, around 5 weeks lymphocyte infiltration is observed (207) and finally after 16 weeks, when obesity is well-established, macrophages become the main infiltrating cells (206, 207). The lymphocyte and macrophage infiltrates in WAT of obese individuals (mice and humans) are mainly pro-inflammatory, while those in the WAT of lean individuals are anti-inflammatory (207–210). Thus, this inflammation has been interpreted as a response of the organism to an insult, which in this case is nutritional energetic overload, and whereby the stimulus persists and inflammation becomes chronic (211, 212). In addition, the diets that are usually consumed in middle- and high-income countries, where there is higher prevalence of obesity, are often unbalanced with very low fruit and vegetable consumption and with excessive meats and ultra-processed products that impose a burden of saturated fats and refined sugars (196, 213–216). Thus, the disturbed energy balance in favor of fat accumulation, that is obesity, leads to a WAT growth with hypertrophy and death of adipocytes promoting macrophage infiltration to phagocytize the remains; these in turn produce pro-inflammatory cytokines that favor further infiltration (217, 218). Saturated fatty acids directly stimulate differentiation to a pro-inflammatory macrophage profile while monounsaturated and polyunsaturated fatty acids promote an anti-inflammatory profile (219). Thus, if the stimulus persists, that is, if the diet is still rich in saturated fatty acids and energy, a vicious circle is established with continuous infiltration of pro-inflammatory macrophages, which maintains the inflammation chronically (220). In addition, while eicosanoids derived from ω-6 polyunsaturated fatty acids, prostaglandins, and leukotrienes, facilitate the initiation of the inflammatory response, the eicosanoids and docosahexanoids derived from ω-3 polyunsaturated fatty acids, resolvins, and protectins, are essential to finishing it (139, 221). It has been found that the diets of obese individuals are usually deficient in ω-3 fatty acids but not in ω-6 fatty acids, and this could contribute to maintaining the chronic inflammation (205, 221, 222). Besides, the hypertrophy of WAT also elicits tissue hypoxia, which favors inflammation and angiogenesis that are needed to expand WAT (209, 223). Therefore, to enable the adipocytes to hypertrophy, the MMPs must break down the extracellular matrix. Accordingly, in a mouse MMP14 null model, researchers observed a reduction in adipose tissue mass and smaller adipocytes (224). Moreover, in obese mice fed an HFD, increased HIF-1α and VEGF levels have been found, and HIF-1α overexpression was causally implicated in obesity-induced insulin resistance (221). Related to inflammation, the NF-κB signaling pathway is also activated in obesity and is responsible for the production of pro-inflammatory cytokines such as TNF-α that induce insulin resistance (139).

Type 2 diabetes is characterized by insulin resistance and is prevalent among people older than 40 years and normally associated with obesity. However, type 2 diabetes has been increasingly described among children and adolescents, related to the change in lifestyle and nutrition (222, 225, 226). Insulin resistance could be promoted through WAT inflammation generated in obesity and liver lipid accumulation (197), which is associated with nutritional energetic overload (225, 227). In addition, the diabetic state also increases liver inflammation with a pro-inflammatory profile of Kupffer cells and the secretion of pro-inflammatory cytokines that aggravate insulin resistance (228). Elevated circulating glucose levels interact with proteins and lipids which are glycated. The process occurs through the Maillard reaction, a complex sequence of chain reactions that leads to the formation of stable advanced glycation end products (AGEs) which are oxidants and can damage tissues (229, 230). AGEs may produce ROS, bind to specific cell surface receptors (RAGE) and contribute to an inflammatory state in diabetes. The interaction between AGE and endothelial RAGE leads to NF-κB upregulation, which transcribes its target genes such as vascular cell adhesion molecule-1 (*VCAM-1*), intercellular adhesion molecule-1 (*ICAM-1*), and *VEGF*; pro-inflammatory cytokines as IL1α, IL6, and TNFα; and RAGE itself (229, 231). The insulin resistance present in diabetes also promotes an increase in the level of circulating lipids (232) due to slow VLDL emptying since the level of lipoprotein lipase is diminished. In addition, there is reduced LDL clearance; consequently, their circulation time is increased, leading to hyperlipidemia and increasing their susceptibility to oxidative modifications (233). In LDL samples obtained from diabetic individuals, there have been observed significantly elevated levels of both apoB and lipid linked to AGE (233). Besides, it was found that macrophages take up the glycated LDL to a greater extent than native LDL, resulting in the formation of foam cells as is characteristic of the early atherosclerotic lesion (234). Thus, the formation of AGE in a hyperglycemic environment contributes to the vascular pathophysiology, promoting the development of atherosclerosis in diabetes (235). The atherosclerotic process also involves an inflammatory process, in which NF-κB plays a major role, contributing to the overall incidence of inflammation in MetS (236).

In addition to comorbidities that exist among obesity, type 2 diabetes, and atherosclerosis, obesity is associated with a higher risk of certain cancers, such as those of the pancreas, liver, colon and rectum, kidney, endometrium, and postmenopausal breast (237). Another condition that also occurs as a comorbidity to MetS is Alzheimer's disease [characterized by chronic brain inflammation in which diet is the risk factor (238–240) and represents the third leading cause of death in the highest income countries] (198).

#### Food Consumption Habits Associated to the Frequency of Metabolic Syndrome

Diverse studies have put in evidence regarding the relationship between alimentary habits and obesity prevalence and, in turn, their comorbidities in various countries such as Brazil, Canada, United States, Australia, and various European countries (241–246). These studies showed a trend in the last decades toward a decrease in the consumption of fresh food (mainly vegetables and fruits) in favor of ultra-processed foods (247) and how this change correlates with the increase in obesity prevalence worldwide. Other studies have correlated the consumption of ultra-processed foods with the increase in sugars, saturated and trans-fat, and Na intake, that in addition, correlate with an associated higher energy intake and obesity prevalence (248–251). In relation to the increase in cancer incidence associated with MetS, Fiolet et al. found that a 10% increase in the proportion of ultra-processed foods in the diet was associated with a significant increase (>10%) in the risk of overall cancer and more specifically of postmenopausal breast cancer, the most commonly related to obesity (237). In summary, all the scientific and epidemiological data lead us to a common point. That is, when diet is unbalanced with energy excess due to high intake of sugar and saturated fat, and with a shortage of fruit and vegetables, the MetS and comorbidities occur.

### Effects of Cruciferous Glucosinolate-Derived Isothiocyanates and Indoles on Metabolic Syndrome

The effects of ITCs and indoles on MetS components have also been studied (252–258). The oxidative stress that exists in MetS, as a consequence of an overload of the homeostatic system, results in pro-inflammatory adipokine secretion, activation of the immune system, and chronic inflammation. The cellular mechanisms of defense against oxidative stress are orchestrated by the transcription factor Nrf2, as stated previously, making it a key target for the amelioration of MetS. Previous data have shown that ITCs and indoles activate Nrf2; thus, they are good candidates for further study. In mice fed an HFD supplemented with glucoraphanin (a GLS precursor of SFN), Nagata et al. (259) found a mitigated weight gain, attenuated fat depot (WAT), decreased hepatic steatosis, and improved glucose tolerance and insulin sensitivity. All these changes occurred without changes in intake but with an increase in energy expenditure. In addition, the increased energy expenditure occurred through WAT browning. The researchers also showed that decreased activation of NF-κB and JNK increased pAkt expression, together with diminished pro-inflammatory macrophage infiltration in WAT. Finally, they also found changes in microbiota with decreased relative abundance of Gram-negative bacteria that was correlated with diminished circulating LPS levels. Glucoraphanin supplementation had no effect in HFD fed Nrf2 knockout mice, suggesting that the anti-obesity effects of glucoraphanin (SFN) are due to the activation of the Nrf2 pathway. Similarly, using an adipocyte (3T3-L1)-macrophage (RAW264.7) co-culture system, Kang et al. described that brassinin (an indole) suppressed inflammation through Nrf2-HO-1 signaling pathway activation (260).

Several other authors described that SFN, AITC, BITC, PEITC, and indoles inhibited adipocyte differentiation in 3T3-L1 cells (261–265) and in mice (265–268). However, recently, Yang et al. described that the same doses of SFN and SFEN (sulforaphene) yielded stronger effects than AITC, BITC, or PEITC in inhibiting the differentiation of 3T3-L1 adipocytes. SFEN was also efficient in inhibiting adipogenesis in human adipose tissue-derived stem cells. The SFEN effect occurred when added early in differentiation and was mediated by decreased C/EBPβ stability, lowering PPARγ and C/EBPα expression (261). These results are in accordance with those reported previously by Chen et al. who showed that adipogenesis inhibition occurred in 3T3-L1 adipocytes through uptake of SFEN via the Hedgehog signaling pathway (263). Earlier, in 3T3-L1 preadipocytes, Choi et al. found that SFN arrested the cell cycle in the G0/G1 phase by increasing the expression of *p27* and decreasing the expressions of cyclin D1, CDK4, cyclin A, and CDK2 (262). In the same study, the authors also found that SFN decreased the phosphorylation of ERK1/2 and Akt. Similar results were obtained by Choi et al. when 3T3-L1 preadipocytes were exposed to I3C; in this case, the cells were arrested in the transition from the S to the G2/M phase together with an increase in p27 expression and a diminished cyclin A expression (269). They also found that inhibition of adipogenesis, similar to that observed with SFEN, was achieved at an early stage of differentiation, and I3C regulates lipid synthesis via AMPKa signaling with diminished ERK and Akt phosphorylation. Yan et al. found that DIM arrested 3T3-L1 proliferation at the G0/G1 phase, but no effect on I3C was found. Moreover, the researchers also concluded that the inhibition of adipogenesis is mediated by targeting USP2 activity (265). In addition, the activation of apoptosis in 3T3-L1 adipocytes was found for SFN through downregulation of the Akt/p70s6k1/Bad pathway and upregulation of the ERK pathway as reported by Yao et al. (270).

In mice fed with an HFD to induce obesity, Choi et al. found that SFN prevents an increase in body weight without changes in food intake. SFN treatment also decreased circulating levels of leptin and cholesterol and increased the level of adiponectin. Fat deposition decrease in WAT takes place through inhibition of C/EBPα and PPARγ and the suppression of lipogenesis via activation of the AMPKa pathway (268). In HFD fed mice, Chuang et al. described that BITC and PEITC prevent body weight gain in a dose-dependent manner with diminishing adipogenesis and prevention of hepatosteatosis through inhibition of the lipogenic regulatory transcription factors PPARγ, LXRα, and SREBP1c, and a decrease in their regulated downstream enzymes (266). They also found that BITC and PEITC had similar effects and that 3T3-L1 cells were arrested in the G0/G1 phase. Similar results have been described for AITC also in HFD fed mice; in this case, the authors found prevention of body weight gain, decreased fat deposition in WAT and liver, and diminished inflammation. Diminished liver lipogenesis was mediated through activation of Sirt/AMPKa signaling, downregulation of SREBP1c, and upregulation of PPARα (271). They also investigated the decreasing liver inflammation and found diminished levels of TNFα, IL1β, and IL6 together with the activation of NF-κB signaling. Treatment of HFD fed mice with I3C (267) or DIM (265) also resulted in a decrease in body weight consequent to fat mass reduction and lipogenesis inhibition. Yagi et al. described that PEITC reduces food intake by activating leptin signaling via hypothalamic leptin receptors (Ob-Rb) and the Janus kinase 2 signal transducer (272). Furthermore, among mice, it has been found through the intraperitoneal glucose tolerance test that AITC reduces hyperglycemia and increases exogenous glucose consumption, ameliorating insulin sensibility (273). Ameliorated insulin sensibility was also found for BITC, PEITC, and AITC in HFD fed mice (266, 274). Jayakumar et al. found that I3C and DIM treatment of HFD fed mice induces decreases in the levels of glucose, insulin, and glycated hemoglobin and ameliorated overall oxidative stress (275). It has recently been reported that SFN could prevent Alzheimer progression and cerebral ischemia (276). Thus, all these pieces of evidence highlight that the use of GLS derivatives from cruciferous vegetables might be considered for the treatment of MetS.

Human studies aimed at investigating the effect of ITCs and indoles on MetS are scarce. Treatment of 40 hypertensive individuals (without diabetes and with normal levels of cholesterol) with 10 g of dried broccoli sprouts during a 4-week period failed to improve endothelial function (277). Another clinical trial examined 81 patients randomized to three groups: one to consume 10 g/d of broccoli sprouts powder (BSP), the other to consume 5 g/d of BSP, and another to consume the placebo for 4 weeks. The results showed that only the consumption of 10 g/d BSP resulted in a significant decrease in serum insulin concentration and HOMA-IR with improved insulin resistance (278). Japanese males with hepatic abnormalities treated with broccoli sprout extract capsules for 2 months showed significantly decreased serum levels of liver function markers (ALT, γGTP, and alkaline phosphatase activity); no changes were observed in the placebo group (279). A controlled trial conducted among healthy men analyzed the potential effect of 10 g freeze-dried nasturtium leaf (rich in BITC) administration on the levels of certain gut hormones that regulate food intake and satiety. The patients avoided the intake of cruciferous vegetables for a 1 week before the intervention. The results showed that the levels of peptide YY (PYY), an anorexigenic gut hormone, were increased after intake of freeze-dried nasturtium leaf during 6 h, and their effect could be more or less depending on whether they carried a polymorphism of the bitter taste receptor TAS2R38. Obese individuals frequently have lower levels of PYY and bariatric by-pass surgery results in elevated PYY levels. Therefore, administration of extract nasturtium (or BITC) or special diets containing nasturtium might be considered in the treatment of obesity (280). A randomized trial of 11 healthy individuals (www.controlled-trials.com ISRCTN19147515) assessed the effect of a dose of mustard (rich in AITC) on energy expenditure during 150 min. The results failed to show any relevant thermogenic response at the highest tolerable dose (281). Finally, López-Chillón et al. conducted a clinical trial of 40 healthy overweight subjects treated with broccoli sprouts over a long period (ClinicalTrials.gov ID NCT 03390855). The treatment phase consisted of 30 g/day of broccoli sprouts consumed for 10 weeks followed by a phase of 10 weeks of normal diet without consumption of these broccoli sprouts. The results showed a positive effect on inflammatory parameters with a significant decrease in IL6 levels that was maintained after treatment. In addition, the levels of GLSs, ITCs, and their metabolites (GRA, IB, SFN, SFN-GSH, SFN-NAC, SFN-CYS, I3C, and 3,3-DIM) were determined in urine as a control of broccoli intake, increased significantly during the treatment period, and decreased thereafter (282).

**Figure 5:**
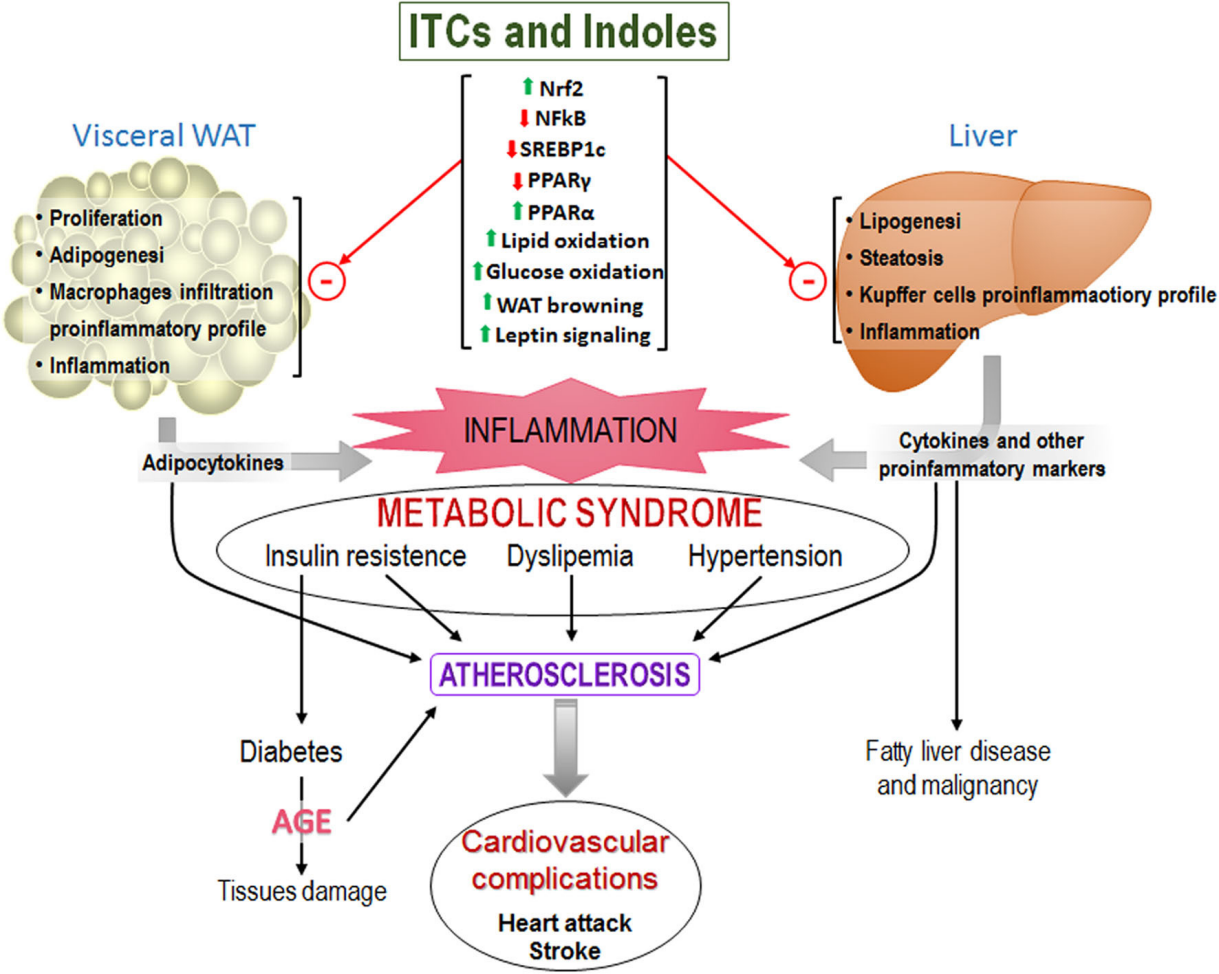
Representative scheme of the main components of the MetS that ultimately lead to cardiovascular problems and others. Upper of the figure are shown the effects of isothiocyanates that have been found and that could prevent MetS development.

Figure 5 is a schema of how the increase in WAT size results in increased infiltration of pro-inflammatory macrophages and secretion of pro-inflammatory cytokines. High energy intake also promotes lipid deposition and inflammation in the liver. Both contribute to systemic inflammation that results in insulin resistance, dyslipidemia, and hypertension (i.e., MetS) which are the main risk factors for cardiac diseases. It shows also the described effects of ITCs and indoles that improve MetS.

## Conclusions

In this review, the important regulatory biological role of cruciferous GLSs derivatives (ITCs and indoles) has been highlighted. Many scientific studies have put in evidence that GLSs derivatives play a key role in regulating central cell pathways and have epigenetic effects. For this reason, GLSs derivatives have been proposed as a possible co-therapy for the treatment of certain cancers, given their activity in the cell cycle, apoptosis, and angiogenesis regulation. More recently, they have been proposed as a possible mechanism in the prevention and/or therapy of MetS or disorders afflicting the central nervous system due to their anti-inflammatory effects. The results in animal and cell studies in relation to the MetS have shown that GLSs derivatives (ITCs and indoles) treatment reduces fat deposition in WAT and liver, decreases proliferation and differentiation of adipocytes, promotes WAT browning with an increase of energy expenditure, improves insulin sensitivity, reduces inflammation and decreases food intake. These results are promising to GLSs derivatives as a possible treatment to prevent MetS, however, there are few human trials. In addition, caution should be exercised since certain toxic effects have also been described (276). When other vegetable components with a high antioxidant capacity, like β-carotenes and vitamin E, have been administered as co-treatment or preventive agents for certain cancers, adverse effects have been found, reaching the point of having to stop the study because cancer progressed faster than among those that did not receive the supplement (283, 284). It is for this reason (without discarding the therapeutic application and the given scientific evidence regarding the important biological role of GLS derivatives in regulating key cellular pathways) that the consumption of cruciferous vegetables, and vegetables in general, in the diet should be claimed. There is a need to return to a healthier diet in which the pyramid base must constitute vegetable foods, of which cruciferous vegetables are essential.

## Author Contributions

The author confirms being the sole contributor of this work and has approved it for publication.

## Conflict of Interest

The author declares that the research was conducted in the absence of any commercial or financial relationships that could be construed as a potential conflict of interest.
